# Knowledge, Attitude, and Practice towards Prevention of Intestinal Helminth Infection among Residents of the Ho Municipality in the Volta Region of Ghana

**DOI:** 10.1155/2023/5515603

**Published:** 2023-12-09

**Authors:** Verner N. Orish, Moses A. Asumbono, Isaac B. Addei, Moses A. Ayaaba, Precious K. Kwadzokpui, Aleksandra Marinkovic, Stephanie Prakash, Chuku Okorie, Ricardo Izurieta, Rajashree Pandit, Adekunle Sanyaolu

**Affiliations:** ^1^Department of Microbiology and Immunology, School of Medicine, University of Health and Allied Sciences, Ho, Volta Region, Ghana; ^2^School of Medicine, University of Health and Allied Sciences, Ho, Ghana; ^3^Department of Medical Laboratory Sciences, School of Allied Health Sciences, University of Health and Allied Sciences, Ho, Ghana; ^4^Laboratory Department, Ho Teaching Hospital, Ho, Volta Region, Ghana; ^5^Saint James School of Medicine, Anguilla; ^6^Union County College (Plainfield Campus), Plainfield, NJ, USA; ^7^Global Communicable Diseases, College of Public Health, University of South Florida, Tampa, Florida, USA; ^8^Universidad de las Américas, Ecuador; ^9^D'Youville University, Buffalo, NY, USA

## Abstract

**Background:**

This study investigated the knowledge, attitude, and practices of residents in the Ho municipality towards intestinal helminth infections and their prevention.

**Method:**

A descriptive cross-sectional study involving questionnaire administration was conducted among randomly sampled residents of the Ho municipality of the Volta Region of Ghana. A minimum sample size of 400 was calculated for subjects using Yamane's formula for population. Pearson's chi-square analysis was done to determine any relationship between sociodemographic characteristics and the categories of knowledge, attitude, and practices. Results from data analysis were computed as frequencies and percentages. *P* < 0.05 is considered statistically significant.

**Results:**

Of the 400 participants, 343 (85.7%) were aware of intestinal helminths, but the majority had poor knowledge of its cause despite 50.5% (202) having good knowledge scores. Most participants (331, 82.8%) had a bad attitude toward preventing the disease. Most (335, 83.8%) of the community members still adhered to the poor practice of open defecation with the excuse of unavailable latrines, and some (143, 35.8%) believe that intestinal helminths are nothing to worry about.

**Conclusion:**

Health education should address the perceptions towards preventing intestinal helminthiasis and be complemented by making available public lavatories in the municipality to curb the unacceptable practice of open defecation.

## 1. Introduction

Intestinal helminths are worm-like macroscopic parasite, whose adult worm inhabits the intestines of humans. They include soil-transmitted helminths which are basically round worms like *Ascaris lumbricoides*, hookworm, and *Trichuris trichiura* [1]. Other intestinal helminths include common cestodes like *Taenia solium*, *Taenia saginata*, and *Diphyllobothrium latum* [1].

In 2022, the Centers for Disease Control and Prevention estimated that between 576 and 740 million people are infected with hookworm, approximately 807-1,121 million people with *Ascaris*, and approximately 604-795 million people with *Trichuris trichiura* [3], with the prevalence of hookworm, *Ascaris lumbricoides*, and *Trichuris trichiura* in sub-Saharan West Africa being described to be 10%, 15.9%, and 5.5%, respectively [4]. Many of these infections are among children, where 880 million children are reported to require treatment for intestinal helminths worldwide [5]. In all, about 24% (more than 1.5 billion) of the world population are infected with intestinal helminth with most of these infections occurring in poor developing countries in South America, East Asia, and sub-Saharan Africa [5].

Pervading poor socioeconomic situation is the hallmark feature common to these areas of the world where intestinal helminth infection is endemic, and it is characterised by poor sanitation, inadequate potable water, and poor handling of human faeces [6]. Another common feature of these areas is poor prevention practices and other practices that encourage and facilitate the transmission of intestinal helminths [7]. These practices are usually influenced by the knowledge and attitude of the people living in these endemic areas, towards intestinal helminths and its prevention.

In Ghana, intestinal helminth infection is common, and many studies have established this fact among pregnant women, children, and food vendors [8]. Studies have reported varied prevalence of intestinal helminth infections among children in the region from a high prevalence of 44% in the Oti region (formally part of the northern part Volta Region) to as low as 2% and below 1% in some parts of the southern part of Volta including the Ho municipality [9–11]. The periodic deworming exercise being carried out among children in some districts in the country is a very significant prevention strategy. It is equally important to constantly assess the prevention practices and attitude of individuals, as well as their knowledge of the disease, as these can influence the transmission of intestinal helminth infections especially among vulnerable persons such as children in the populations [11]. This study thus assesses the knowledge, attitude, and practices towards intestinal helminth infection prevention among residents in the Ho municipality, Volta Region of Ghana.

## 2. Materials and Methods

### 2.1. Study Site

**Figure 1:**
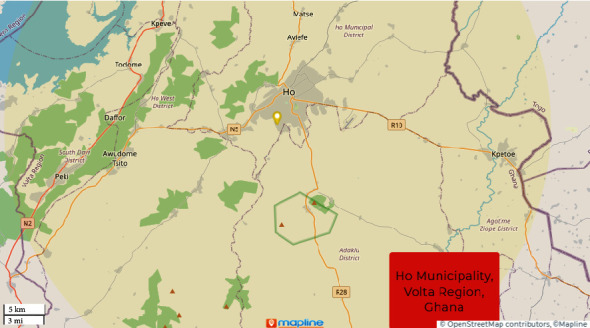
Map of Ho municipality.

This study was carried out in the Ho municipality of the Volta Region of Ghana, West Africa (Figure 1). The Ho municipality lies between latitudes 6° 207 N and 6° 55 N and longitudes 0° 127 E and 0° 53 E and covers an area of 587 sq. km. The municipality shares boundaries with the Adaklu-Anyigbe District to the South, Hohoe Municipal to the North, South-Dayi District to the West, and the Republic of Togo to the East [12]. The Ho municipality is the administrative and regional capital of the Volta Region of Ghana consisting of fifty-eight electoral areas. According to the 2020 population and housing census, the Ho municipality has a population of 180,420 representing 10.9 percent of the region's total population with 84,843 males representing 47.0 percent and 95,577 females representing 53.0 percent of the population [13]. Households in this municipality get drinking water from different sources, including standpipes, borehole, tube wells, and rivers or streams, with most of them (58%) using pipe-borne water and as high as 7.4% using rivers and streams. For other domestic use, 53% and 10% use pipe-borne water and rivers or streams, respectively. Also, 16.4% of households of Ho municipality lack toilet facilities and report using open defecation in the field or bush. Concerning waste management, 29% of residents use open dumpsites while 4.4% practice indiscriminate disposal of solid waste [14]. About 35% of the households in the Ho municipality are involved in agricultural activities.

### 2.2. Study Population

This study was done among adult residents of the Ho municipality aged 15 and above who have lived in the municipality for at least one year.

### 2.3. Sample Size Determination

A minimum sample size of 400 was calculated using Yamane's formula for population *n* = *N*/(1 + *N*(*e*)^2^), estimating for a tolerable error of 5%, with a confidence interval of 95%, where *n* is the sample size, *N* is the study population = 180,420 (population of Ho municipality 2021 census) [15], and *e* = 5% (0.05) suggested margin error. This resulted to 399.1 and subsequently approximated to 400 minimum sample size.

### 2.4. Study Design and Sampling Technique

This is a descriptive cross-sectional study that measured the knowledge, attitude, and practice towards intestinal helminth infections among residents of the Ho municipality of the Volta Region. Residents were randomly sampled from strategic locations in the municipality so that residents might have a fair representation. Churches and mosques were visited, likewise salons and marketplaces as well as supermarkets, shopping malls, and other workplaces. Only those above the age of 15 years and who are residents of the municipality for at least 12 months were recruited. Conversely, those passing by the municipality especially the market folks or those who work in the municipality but reside outside it were excluded from recruitment.

### 2.5. Questionnaire Development and Validation

The questionnaire used in this study was solely developed for the purpose of obtaining the data used in this study. Subsequently, a face validity of the questionnaire was carried out through the expert review of two independent public health specialists. After the reviews of the questionnaire with some minor alterations of the questions, the experts adjudged the questionnaire to be of reasonable constructs and considered it for purpose. The English language was the language of choice as it was considered that the majority of the participants in the municipality can read and understand English, and if there were any participants who do not understand, the questions were interpreted in the language they understand.

### 2.6. Data Collection Procedure

The questionnaire was divided into 4 sections: (1) sociodemographic characteristics, (2) level of knowledge of helminth infections, (3) attitude towards helminth infections, and (4) prevention practices of intestinal helminth infections as well as its barriers. Information on the sociodemographic characteristics of the residents obtained include age, sex, marital status, level of education, occupation, and religion.

Knowledge among the participants was determined by asking questions on aetiology, sign and symptoms, transmission, treatment, and prevention of intestinal helminths. To assess the knowledge on the cause of intestinal worms among the participants, viruses, bacteria, and parasites were described in the simple terms for illiterate participants to understand. Each response was scored as “yes” or “no”/“I don't know.” A correct answer was awarded 1 mark and a zero mark for wrong answer/“I do not know.” There were 51 questions in all; thus, the scoring range was 51 (maximum) to 0 (minimum). Knowledge scores for each question were calculated and summed up to give the total knowledge score. This knowledge score was then computed as percentages and then placed into categories depending on the percentage of knowledge score. A cutoff level of <60% was considered as poor whereas 60% to 79.9% was considered as moderate/good knowledge and 80% to 100% as excellent knowledge about intestinal worms.

For the attitude section, 12 questions were used, and scores of 2 and 1 were given for choosing the answer reflecting a “very” positive attitude and positive attitude, respectively, while an answer reflecting a negative attitude was awarded a zero mark. The maximum expected mark was 24 (100%), and the lowest mark was zero (0%). These were then grouped into bad attitude (0% to 59.9%), good attitude (60% to 79.9%), and very good attitude (80% to 100%).

A total of 13 questions were used to assess various preventive practices with responses like “always,” “occasional,” and “never.” Scores 2 and 1 were given for responses that represent answers for acceptable prevention practices, while score 0 is given for any response that represents an answer for unacceptable practices in the prevention of intestinal worm infection. The maximum scores are expected to be 26 (100%) and the minimum score of 0 (0%). These scores were further computed to find the practice percentages and then graded as acceptable practices (practice score percentage > 80%) and unacceptable practices (practice score percentage < 80%).

### 2.7. Statistical Analysis

The data collected was cleaned, edited, and reorganized to exclude error and imputed into Statistical Package for Social Sciences (SPSS version 24) for analysis. Knowledge was categorised into poor, good, and excellent, while attitude was categorised into bad, good, and very good. Practices that prevent or encourage intestinal helminth infection were categorised into bad, good, and excellent. Results from data analysis were computed as frequencies and percentages, and Pearson's chi-square analysis was performed to determine relationship between sociodemographic characteristics and the categories of knowledge, attitude, and practices. *P* < 0.05 is considered statistically significant.

### 2.8. Ethical Consideration

This study commenced after ethical approval from the University of Health and Allied Sciences ethics committee was obtained (UHAS-REC A.12 [50] 20-21), and an introductory letter from the School of Medicine was sent to the authorities of the municipal assembly for approval to collect the data, and other stakeholders like the chiefs and pastors and imams were informed about the proposed study. Participants were recruited after informed consent was obtained from each participant.

## 3. Results

There was a total of 400 respondents in this study sampled from various communities in Ho municipality. Information collected include sociodemographic characteristics, knowledge level, attitude, and prevention practices towards intestinal helminth infections and its barriers.

### 3.1. Sociodemographic Characteristics

The ages of the respondents ranged from 15 to 65 years, with a mean (SD) age of 28.00 (9.154) years (Table 1). Most of the respondents were females (212, 53.0%), while 188 (47%) were males. Most of the respondents were single (202, 50.5%), while married and divorced/widowed accounted for 187 (46.8%) and 11 (2.8%), respectively. The education levels of the respondents were tertiary (160, 40.0%) followed by secondary school (133, 33.3%). Occupation status of the respondents revealed that 115 (28.7%) were unemployed (students, unemployed people, and housewives), traders and others that own their own business such as shop owners were grouped as self-employed and accounted for 24.5% of the participants, and 82 (20.5%) of the participants were public sector workers like teachers, nurses, and doctors and, therefore, were grouped as civil servants.

### 3.2. Knowledge Level of Intestinal Helminth Infection

A total of 343 (85.7%) participants were aware of intestinal parasites. Some participants wrongly chose viruses (organisms that cause common cold) as the cause of intestinal worms (179, 44.8%), while 137 (34.3%) of them chose bacteria (organisms that cause pus in wound) as the cause of intestinal worms. A total of 231 participants answered correctly that intestinal worms are caused by parasites (small animals that live in the intestines). In terms of transmission, some participants accurately answered that intestinal worms can be transmitted through the ingestion of contaminated water (364, 91%), vegetables, and fruits (350, 87.5%) and also by eating with unwashed hands (333, 83.3%) and eating raw meat or fish (305, 76.3%), and 202 participants accurately answered that walking barefooted can also transmit intestinal worms (202, 60.5%). However, some participants (125, 31.3%) wrongly stated that eating sweets and candies can cause intestinal worms. Some participants had the misconception that intestinal helminth infection is contracted from inhaling contaminated air (170, 42.5%) and touching infected people (169, 42.3%), and 189 (47.3%) participants said that it is contracted from the sweats of infected people. A total of 344 (86%) accurately answered that children are at increased risk of infection. Only 66 (16.5%), 68 (17.0%), and 54 (13.5%) of the participants were aware that intestinal worms could result in pneumonia, breathing difficulties, and bronchitis, respectively. The majority of the participants answered correctly that intestinal worms can be prevented (340, 85%) and are treatable (357, 89.3%) with the majority answering that avoiding open defecation (343, 85.7%) is a way to prevent intestinal worm infections, and dewormers (357, 89.3%) can treat intestinal worms (Table 2).

**Figure 2:**
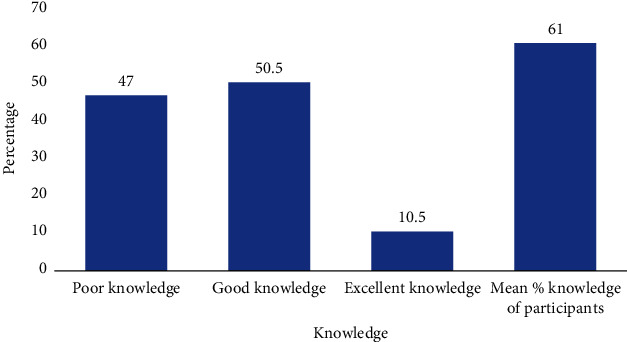
Percentage categories of participants on knowledge.

Out of the 400 participants, 188 (47.0%), 202 (50.5%), and 10 (2.5%) of the participants were within the poor knowledge (<60% score), moderate/good knowledge (60-79% score), and excellent knowledge (80-100% score) range, respectively. The mean score for knowledge among study participants was 61.0% (Figure 2).

### 3.3. Attitude towards Intestinal Helminth Infection

A total of 189 (47.2%) and 143 (35.8) did not agree that intestinal worms cause serious illness and there was no need to be worried about it, respectively. Herbal medication was considered an effective treatment by 327 (81.7%) participants while 124 (31.0%) did not consider dewormers as effective treatment for intestinal worms. Some (143, 35.7%) of the participants believe that intestinal worms' infestation is not a problem in Ghana, and a significant percentage (154, 38.5%) believe that it cannot cause growth retardation in children. Some also opined that if they see worms in their stool, they will do nothing about it (100, 25.2%) (Table 3).

**Figure 3:**
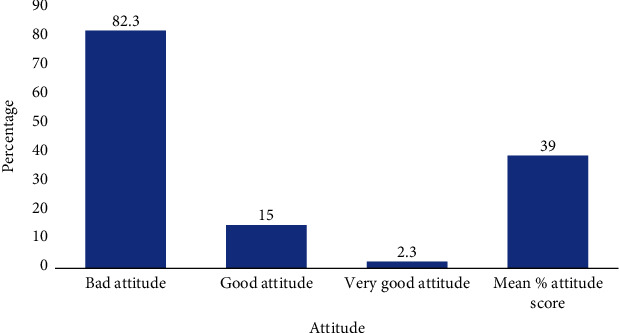
Percentage categories of participants on attitude.

Most of the residents had a bad attitude (331, 82.8%) towards the prevention of intestinal helminth infections, while quite a few (60, 15.0%) of the participants had a good attitude, and 9 (2.3%) of the participants had a very good attitude towards intestinal worm prevention. The mean (SD) of the attitude percentage scores was 39% (Figure 3).

### 3.4. Preventive Practices against Intestinal Helminths

Most of the participants occasionally eat unwashed vegetable and fruits (48.0% and 53.3%, respectively). Some occasionally eat raw meat (30, 7.5%) and fish (48, 12.0%). However, more than half of them practiced protective practices, including hand washing after using the toilet, and before and after eating, but some of them practice open defecation (always 45, 11.2%; occasionally 113, 28.3%). Walking on bare feet outside the compound (always 34, 8.5%; occasionally 272, 68.0%) and in the farm (always 78, 19.5%; occasionally 120, 30.0%) was fairly a common practice among participants. About 71(17.8%) of the participants reported to have never dewormed before (Table 4).

**Figure 4:**
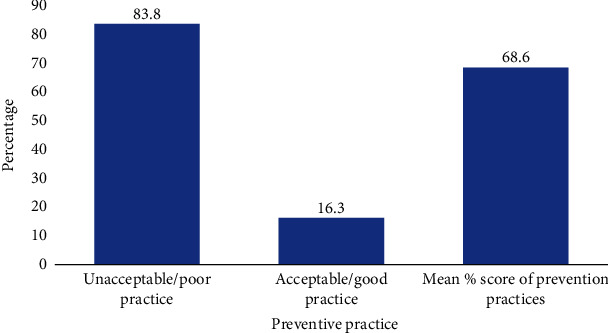
Percentage categories of participants on preventive practices.

Among the participants, 65 (16.3%) had a good practice, and 335 (83.8%) had a poor practice towards intestinal helminth infection prevention as represented on the bar chart below, and the mean percentage score of the participants was 68.6% (Figure 4).

### 3.5. Sociodemographic Associations between Knowledge, Attitude, and Preventive Practices

The age of the participants had no significant association with attitude and prevention practices but was associated with knowledge. Participants below the age of 30 years had the highest proportion of those with poor level of knowledge (123, 65.4%, *p* = 0.009). Occupation had a significant association with knowledge and attitude with participants who are civil servants proportionally having the highest percentage of excellent knowledge (6, 60%, *p* = 0.007) and a very good attitude (6, 66.7%, *p* = 0.002). Education also had a significant association with knowledge and attitude with participants who had tertiary education proportionally having the highest percentage of excellent knowledge (8, 80%, *p* = 0.001), and very good attitude (8, 88.9%, *p* ≤ 0.001). Marital status was only significant with prevention practices, with single participants proportionally having the highest percentage of acceptable prevention practices (41, 63.1%, *p* = 0.046) (Table 5).

Table 6(a) shows the association between level of knowledge and attitude and practices. Level of knowledge was significant with attitude (*p* = 0.013). Those with poor knowledge had the highest number of participants with bad attitude (167, 50.5%, *p* = 0.013).

Table 6(b) shows a significant association between attitude and practice (*p* = 0.005). Participant with bad attitude had the highest unacceptable practice (279, 83.3%, *p* = 0.005).

## 4. Discussion

The respondents in this study had a moderate/good level of knowledge (50.5%), a mean score of 61% with 85.7% of the participants being aware of intestinal helminth. This is consistent with studies conducted within communities in Nigeria [16] and Zimbabwe [17], where more than 85% expressed their awareness. We could attribute this to the frequent health talks within the communities where intestinal helminths are endemic [18]. Although the respondents' awareness corresponds with similar studies conducted by Oguh *et al*. and Aribodor *et al*., there were still lapses in their knowledge on the aetiology as a significant number of the participants wrongly answered that eating candy causes intestinal helminth infection [16, 19]. In as much as 40% of the respondents in this study had received tertiary education, their knowledge on the aetiology of intestinal helminths did not match their awareness, unlike as reported in the Sam-Wabo *et al*. [20] study where tertiary students also had an excellent knowledge on its aetiology. Like other studies by Abe *et al*. and Schmidlin *et al*., participants did show excellent knowledge on the mode of transmission and preventive practices probably because the health education information in the Ho municipality may have been focused on that area [17, 21].

Even though the study participants were knowledgeable on the treatment modalities and best preventable practices, the majority (82.8%) had a bad attitude towards its prevention. Fewer participants consented to seeking medical care while the majority did not see it as a problem. This perspective of underestimating the impact of intestinal helminth infection could stem from the neglect of continuous education and community sensitization, as attention is shifted to the COVID-19 pandemic [22]. Also, most community members preferred herbal medications for treatment as this is common in the part of the continent. Acka *et al*. established that indigenes in a local community in Cote d'Ivoire perceived that traditional medicine is effectively cheaper for treating intestinal helminth infection [23], but community health workers (CHW) and school teachers in Rwanda think otherwise [24]. However, their view of choosing herbal medication over dewormers could be because they do not recognize intestinal helminthiasis as a problem for adults; hence, children should be those considered for deworming [24]. Additionally, even though Ghana produces quality antihelminthics [25], most patients in the tropics and particularly from Africa belong to low socioeconomic groups and cannot afford to buy expensive medicines thereby opting for cheaper alternative traditional medicines [25]. In addition, consumers may lack trust on the efficacy of the anthelminthic drugs sold on the market. Herbs have been used to treat intestinal parasites; however, some have toxic side effects or can interfere with other medications. *Carica papaya* is a fruit plant also called papaya, papaw, pawpaw, mamao, or tree melon, found virtually in every tropical and subtropical country that contains proven anthelminthic properties [26].

As expected, participants with tertiary education put up a better attitude towards intestinal helminth infection, as findings in this study highlighted the influence of educational status on the attitude of the participants (8, 88.9%, *p* ≤ 0.001) because of their excellent knowledge (8, 80%, *p* = 0.001). Similar studies conducted in Nigeria [19], Rwanda [24], Tanzania [18], Bangladesh [27], and India [28] asserted the above.

The findings from this study also showed poor preventive practices with a mean percentage score of 68.6% (unaccepted practice < 80%) as most of the participants occasionally walked barefooted, ate unwashed fruits, and still practiced open defecation. These practices might be difficult to change in the absence of basic amenities. For instance, in western Cote d'Ivoire, a similar study agreed that the abundant knowledge and awareness created on intestinal helminths will not be enough to reform behavioural changes when the community has no access to potable drinking water and public latrines [21]. In the absence of these, indigenes will still use the open defecation method and drink and wash their fruits from unhygienic water sources thereby increasing the prevalence of intestinal helminth infection [16]. On the contrary, the societal status and living conditions of civil servants (teachers, nurses, and doctors) may prevent their engagement in such bad practices [29]. In the case of the municipality, open defecation is still a problem as depicted by our study findings probably fuelled by the lack of latrines in some households and few public toilets available in the municipality [13, 30].

One unexpected finding in this study is that individuals who are married in Ho municipality (low-income area) [31] are likely to engage in unacceptable practices. It is not an unusual practice for them to allow their little children to openly defecate, probably because there are no latrines [24] or the latrines available may be too risky for their use. Also, the increased financial burden on the parents to provide sanitary items and healthy meals may have contributed to the problem [32].

The limitation of this study is that participants were administered only a structured questionnaire to ascertain their knowledge, attitude, and practices. This could subject the findings to recall bias especially when practices were not observed. A focus group discussion especially among individuals that practice open defecation or those involved in other negative practices can increase the objectivity of our findings. Despite this, interesting findings that add to knowledge, attitude, and practices towards preventing and mitigating the burden of intestinal helminths in the community were reported, and this information need to be further investigated with more detailed and advanced studies.

## 5. Conclusion

This study shows that the majority of the participants underestimated intestinal helminth infection because they had lapses in the knowledge of its aetiology despite being aware of its existence and mode of transmission. However, this poor knowledge influenced their poor attitude towards its prevention with most of the respondents still treating the infection with traditional medicines perhaps due to their low socioeconomic status. We recommend that health education should be targeted at addressing the perceptions towards preventing intestinal helminthiasis and complemented with making available public latrines in the municipality to curb the negative behaviour of open defecations.

## Figures and Tables

**Table 1:** Sociodemographic characteristics.

Sociodemographic	Variables	Frequency	Percentage
Age	<30 years	233	58.3
	30-40 years	122	30.5
	41-50 years	33	8.3
	51-60 years	8	2.0
	>60 years	4	1.0

Gender	Male	188	47.0
	Female	212	53.0

Marital status	Single	202	50.5
	Married	187	46.8
	Divorced	11	2.8

Religion	Christian	284	71.0
	Muslim	83	20.8
	African traditional religion	27	6.8
	Others (none)	6	1.5

Educational level	No formal education	18	4.5
	Primary	26	6.5
	Junior high school	63	15.8
	Senior high school	133	33.3
	Tertiary	160	40.0

Occupation	Unemployed	115	28.7
	Civil servants	82	20.5
	Self-employed	98	24.5
	Artisans	51	12.8
	Farmers	14	3.5

**Table 2:** Knowledge of participants.

No.	Knowledge	Correct *N* (%)	Wrong/I do not *N* (%)
1	**Have you ever heard of intestinal worm infection?**	343 (85.7)	57 (14.3)
2	**Intestinal worms' infection is caused by**		
I	Organisms that cause common cold/virus	179 (44.8)	221 (55.2)
II	Organisms that cause pus in wounds/bacteria	137 (34.3)	263 (65.7)
III	Small animals that live inside the intestines/parasites	231 (57.7)	169 (42.3)
3	**Intestinal worms spread through**		
I	Contaminated water	364 (91.0)	36 (9.0)
II	Contaminated air	230 (57.5)	170 (42.5)
III	Touching infected people	231 (57.7)	169 (42.3)
IV	Sweat of infected people	211 (52.7)	189 (47.3)
V	Eating with contaminated hands	333 (83.3)	67 (16.7)
VI	Walking barefooted on the open soil	242 (60.5)	158 (39.5)
VII	Eating contaminated fruits and vegetables	350 (87.5)	50 (12.5)
VIII	Eating raw meat and fish	305 (76.3)	95 (23.7)
IX	Eating too much sweets	125 (31.3)	275 (68.7)
4	**Symptoms of intestinal worm infections include**		
I	No symptoms	76 (19.0)	324 (81.0)
II	Headache	222 (55.5)	178 (44.5)
III	Reduce blood level	239 (59.7)	161 (40.3)
IV	Abdominal pain	328 (82.0)	72 (18.0)
V	Anorexia	227 (56.7)	173 (43.3)
VI	Nausea	292 (73.0)	108 (27.0)
VII	Vomiting	339 (84.7)	61 (12.3)
VIII	Diarrhoea	275 (68.7)	125 (31.3)
IX	Constipations	198 (49.5)	202 (50.5)
X	Creepy sensation on the skin	158 (39.5)	242 (60.5)
XI	Easy tiredness	242 (60.5)	158 (39.5)
5	**The following persons are at an increased risk of intestinal worm infection**		
I	Everybody	140 (35.0)	260 (65.0)
II	Pregnant women	219 (54.7)	181 (45.3)
III	Children	344 (86.0)	56 (14.0)
IV	Farmers	286 (71.5)	114 (28.5)
V	People in overcrowded homes	237 (59.3)	163 (40.7)
VI	Very ill people	82 (20.5)	318 (79.5)
VII	The aged (>65 years)	93 (23.3)	307 (76.7)
6	**Is intestinal worm infection preventable**	340 (85.0)	60 (15.0)
7	**Is intestinal worm infection treatable**	357 (89.3)	43 (10.7)
8	**Intestinal worm infection can be treated with**		
I	Painkillers	232 (58.0)	168 (42.0)
II	“Dewormers”	357 (89.3)	43 (10.7)
III	Antibiotics	96 (24.0)	304 (76.0)
IV	Hot foods and liquids	131 (32.7)	269 (67.3)
9	**Intestinal worm infection can be prevented by**		
I	Eating hot foods	35 (8.7)	365 (91.3)
II	Wearing footwear all the time	252 (63.0)	148 (37.0)
III	Washing hands regularly	364 (91.0)	36 (9.0)
IV	Regular exercise	227 (56.7)	173 (43.3)
V	Avoiding open defecation	343 (85.7)	57 (14.3)
VI	Wearing nose mask in crowded places	241 (60.3)	159 (39.7)
VII	Ensuring a healthy diet	343 (85.7)	57 (14.3)
10	**Intestinal worm infection can cause/lead to**		
I	Pneumonia	66 (16.5)	334 (83.5)
II	Difficulty in breathing	68 (17.0)	332 (83.0)
III	Bronchitis	54 (13.5)	346 (86.5)
IV	Intestinal obstruction	274 (68.5)	126 (31.5)
V	Anaemia	289 (72.3)	111 (27.7)
VI	Malnutrition	317 (79.3)	83 (20.7)
VII	Reduced mental development in children	219 (54.7)	181 (45.3)

Bold words represents questions while unbold words represents options to each question.

**Table 3:** Attitude of participants.

No.	Attitudes	Strongly agree	Agree	“Not sure,” disagree, and strongly disagree
1	Intestinal worms do cause serious illness	105 (26.3%)	106 (26.5%)	189 (47.2%)
2	We should be worried about intestinal worm infections	107 (26.7%)	150 (37.5%)	143 (35.8%)
3	Herbal medications are useful in treatment	327 (81.7%)	52 (13.0%)	21 (5.3%)
4	“Dewormers” are effective in treatment	74 (18.5%)	202 (50.5%)	124 (31.0%)
5	Problem for both children and adult	77 (19.3%)	141 (35.3%)	182 (45.5%)
6	Intestinal worm infection is common in Ghana	45 (11.25%)	212 (53.0%)	143 (35.7%)
7	If someone sees worms in his/her stools?			
I	Seek medical care	217 (54.3%)	158 (39.5%)	25 (6.2%)
II	Take herbal preparation	316 (79.0%)	59 (14.7%)	25 (6.3%)
III	Do nothing about it	101 (25.2%)	165 (41.3%)	134 (33.5%)
8	Health education can help	165 (41.3%)	132 (33.0%)	103 (25.7%)
9	Worms can cause growth retardation in children	122 (30.5%)	124 (31.0%)	154 (38.5%)

**Table 4:** Prevention practices among participants.

No.	Practice	Always	Occasional	Never
1	I eat raw unwashed vegetables	33 (8.3%)	192 (48.0%)	175 (43.7%)
2	I eat unwashed fruits	47 (11.7%)	213 (53.3%)	140 (35.0%)
3	I eat raw meat	9 (2.2%)	30 (7.5%)	361 (90.3%)
4	I eat raw fish	13 (3.2%)	48 (12.0%)	339 (84.7%)
5	I do open defecation	45 (11.2%)	113 (28.3%)	242 (60.5%)
6	I use soap to wash my hands after using the toilet	19 (4.7%)	201 (20.3%)	180 (45.0%)
7	I use soap to wash my hands before and after eating	21 (5.2%)	255 (63.7%)	124 (31.0%)
8	I walk barefooted within and outside the compound	34 (8.5%)	272 (68.0%)	94 (23.5%)
9	I allow children to defecate anywhere because children stools are not harmful	23 (5.7%)	68 (17.0%)	309 (77.3%)
10	**In my farming process**			
I	I work with my bare hands and feet	78 (19.5%)	120 (30.0%)	202 (50.5%)
II	I use human excreta for manure or as fertilizer	9 (2.3%)	27 (6.7%)	364 (91.0%)
III	I eat my farm produce while on the farm without washing them thoroughly	25 (6.3%)	131 (32.7%)	244 (61.0%)
11	I deworm myself every three months	116 (29.0%)	213 (53.3%)	71 (17.5%)

Bold words represents questions while unbold words represents options to each question.

**Table 5:** Association between sociodemographic with knowledge, attitude, and practice.

	Poor knowledge (188)	Good knowledge (202)	Excellent knowledge (10)	*p*	Bad attitude (331)	Good attitude (60)	Very good attitude (9)	*p*	Unacceptable practices (335)	Acceptable practices (65)	*p*
*Gender*											
Male	88 (46.8)	96 (47.5)	4 (40)	0.9	160 (48.3)	23 (38.3)	5 (55.6)	0.315	160 (47.8)	28 (43.1)	0.489
Female	100 (53.2)	106 (52.5)	6 (60)		171 (51.7)	37 (61.7)	4 (44.4)		175 (52.2)	37 (56.9)	
*Religion*											
Christian	136 (72.3)	140 (69.3)	8 (80)	0.11	230 (69.5)	46 (76.7)	8 (88.9)	0.738	236 (70.4)	48 (73.8)	0.078
Muslim	32 (17)	49 (24.3)	2 (20)		71 (21.5)	11 (18.3)	1 (11.1)		68 (20.3)	15 (23.1)	
African religion	14 (7.5)	13 (6.4)	0 (0)		25 (7.5)	2 (3.3)	0 (0)		27 (8.1)	0 (0)	
Others (none)	6 (3.2)	0 (0)	0 (0)		5 (1.5)	1 (1.7)	0 (0)		4 (1.2)	2 (3.1)	
*Marital status*					331	60	9				
Single	105 (55.9)	94 (46.5)	3 (30)	0.14	163 (49.3)	34 (56.7)	5 (55.6)	0.527	161 (48.1)	41 (63.1)	0.046^∗^
Married	80 (42.5)	100 (49.5)	7 (70)		160 (48.3)	23 (38.3)	4 (44.4)		163 (48.7)	24 (36.9)	
Divorced	3 (1.6)	8 (4)	0 (0)		8 (2.4)	3 (5)	0 (0)		11 (3.2)	0 (0)	
*Age*											
≤30 years	123 (65.4)	106 (52.5)	4 (40)	0.009^∗^	194 (58.6)	35 (58.3)	4 (44.4)	0.787	188 (56.1)	45 (69.2)	0.226
30-40 years	50 (26.6)	67 (33.2)	5 (50)		101 (30.5)	16 (26.6)	5 (55.6)		105 (31.4)	17 (26.2)	
41-50 years	13 (6.9)	20 (9.9)	0 (0)		26 (7.9)	7 (11.7)	0 (0)		30 (9)	3 (4.6)	
51-60 years	2 (1.1)	6 (3)	0 (0)		7 (2.1)	1 (1.7)	0 (0)		8 (2.3)	0 (0)	
>60 years	0 (0)	3 (1.4)	1 (10)		3 (0.9)	1 (1.7)	0 (0)		4 (1.2)	0 (0)	
*Occupation*											
Unemployed	61 (32.4)	53 (26.2)	1 (10)	0.007^∗^	91 (27.5)	21 (35)	3 (33.3)	0.002^∗^	94 (28.1)	21 (32.3)	0.467
Civil servants	23 (12.2)	53 (26.2)	6 (60)		57 (17.2)	19 (31.6)	6 (66.7)		70 (20.9)	12 (18.5)	
Self-employed	52 (27.7)	45 (22.3)	1 (10)		88 (26.6)	10 (16.7)	0 (0)		84 (25.1)	14 (21.5)	
Artisans	25 (13.3)	25 (12.4)	1 (10)		44 (13.3)	7 (11.7)	0 (0)		40 (11.8)	11 (16.9)	
Farmers	5 (2.7)	9 (4.5)	0 (0)		14 (4.2)	0 (0)	0 (0)		14 (4.2)	0 (0)	
Private employee	22 (11.7)	17 (8.4)	1 (10)		37 (11.2)	3 (5)	0 (0)		33 (9.9)	7 (10.8)	
*Education*											
No formal education	15 (8)	3 (1.5)	0 (0)	0.001^∗^	18 (5.4)	0 (0)	0 (0)	<0.001^∗^	17 (5.1)	1	0.419
Primary	17 (9)	8 (4)	1 (10)		25 (7.6)	1 (1.7)	0 (0)		22 (6.6)	4	
JHS	35 (18.6)	28 (13.9)	0 (0)		61 (18.5)	2 (3.3)	0 (0)		56 (16.7)	7	
SHS	56 (29.8)	76 (37.6)	1 (10)		116 (35)	16 (26.7)	1 (11.1)		111 (33.1)	22	
Tertiary	65 (34.6)	87 (43)	8 (80)		111 (33.5)	41 (68.3)	8 (88.9)		129 (38.5)	31	

*P* < 0.05^∗^.

**(a):** 

Knowledge	Attitude			*p*	Practice		*p*
	Bad (331)	Good (60)	Very good (9)		Unacceptable (335)	Acceptable (65)	
Poor	167 (50.5)	20 (33.3)	1 (11.1)	0.013	154 (45.9)	34 (52.3)	0.56
Good	157 (47.4)	38 (63.3)	7 (77.8)		173 (51.6)	29 (44.6)	
Excellent	7 (2.1)	2 (3.3)	1 (11.1)		8 (2.4)	2 (3.1)	

**(b):** 

Attitude	Practice		
	Unacceptable (335)	Acceptable (65)	*p*
Bad	279 (83.3)	52 (80.0)	0.005
Good	52 (15.5)	8 (12.3)	
Very good	4 (1.2)	5 (7.7)	

## Data Availability

The datasets used during the current study are available from the corresponding author upon request.
